# Incorporation of a Toll-like receptor 2/6 agonist potentiates mRNA vaccines against cancer and infectious diseases

**DOI:** 10.1038/s41392-023-01479-4

**Published:** 2023-07-17

**Authors:** Yangzhuo Gu, Jingyun Yang, Cai He, Tingmei Zhao, Ran Lu, Jian Liu, Xianming Mo, Fuqiang Wen, Huashan Shi

**Affiliations:** 1 Department of Biotherapy, State Key Laboratory of Biotherapy and Cancer Center, West China Hospital, Sichuan University, and Collaborative Innovation Center for Biotherapy, Chengdu, Sichuan 610041 China; 2 Division of Pulmonary Diseases, State Key Laboratory of Biotherapy and Department of Respiratory and Critical Care Medicine, West China Hospital, Sichuan University, Chengdu, Sichuan 610041 China; 3 Laboratory of Stem Cell Biology and Department of Pediatric Surgery, State Key Laboratory of Biotherapy, West China Hospital, Sichuan University, and Collaborative Innovation Center for Biotherapy, Chengdu, Sichuan 610041 China

**Keywords:** Vaccines, Molecular medicine

## Abstract

mRNA vaccines have emerged rapidly in recent years as a prophylactic and therapeutic agent against various diseases including cancer and infectious diseases. Improvements of mRNA vaccines have been underway, among which boosting of efficacy is of great importance. Pam2Cys, a simple synthetic metabolizable lipoamino acid that signals through Toll-like receptor (TLR) 2/6 pathway, eliciting both humoral and cellular adaptive immune responses, is an interesting candidate adjuvant. To investigate the enhancement of the efficacies of mRNA vaccines by Pam2Cys, the adjuvant was incorporated into mRNA-lipid nanoparticles (LNPs) to achieve co-delivery with mRNA. Immunization with the resulting mRNA-LNPs (Pam2Cys) shaped up the immune milieu in the draining lymph nodes (dLNs) through the induction of IL-12 and IL-17, among other cytokines. Antigen presentation was carried out mainly by migratory and dLN-resident conventional type 2 DCs (cDC2s) and significantly more potent antitumor responses were triggered in both prophylactic and therapeutic tumor models in a CD4^+^ and CD8^+^ T cell-dependent fashion. Accompanying memory antitumor immunity was also established. Moreover, the vaccine also stimulated much more robust humoral and cellular immunity in a surrogate COVID-19 prophylactic model. Last but not the least, the new vaccines exhibited good preliminary safety profiles in murine models. These facts warrant future development of Pam2Cys-incorporated mRNA vaccines or relevant mRNA therapeutics for clinical application.

## Introduction

Recent years have witnessed the unprecedentedly rapid development of mRNA vaccines. mRNA vaccines targeting SARS-CoV-2 have experienced an expeditious bench-to-bedside transition, and have made prominent contribution in the fighting against COVID-19 pandemic.^1–4^ Meanwhile, multiple pipelines targeting various cancers and other infectious diseases are also under intense research and development.^5^ mRNA vaccines hold great promise in that mRNA only has to reach the cytosol to exert its function, and does not cause insertional mutagenesis of the host genome. Moreover, mRNA vaccines are tunable transient modulators of physiologic processes, and can be bulk produced within short time in a technically and economically friendly manner.^6–10^

mRNA molecules are typically encapsulated in lipid nanoparticles (LNPs) to form mRNA vaccines. The common components of LNPs are four lipids, namely, an ionizable cationic lipid, DSPC, cholesterol, and a PEGylated lipid. On formulation, hydrophilic and hydrophobic compartments are present in LNPs,^11^ which opens the door to the co-delivery of other molecules. As mRNA vaccines step into the spotlight, its optimization for general use is a field of unremitting research efforts.^12–16^ Efficacy, safety, manufacture-friendliness and cost-efficiency are matters of concern in the optimization process.

**Fig. 1:**
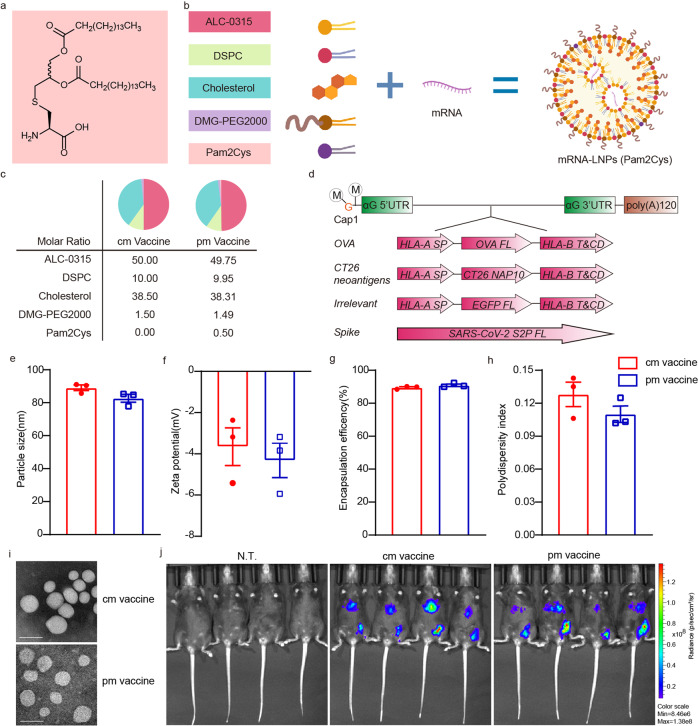
Preparation of Pam2Cys-incorporated mRNA vaccines. **a** Structure of Pam2Cys. **b** Schematic representation of the formulation of mRNA-LNPs (Pam2Cys). This schematic illustration was created by Biorender. **c** The lipid composition of cm vaccine and pm vaccine. **d** mRNAs used in this study. M in circle, methyl group in Cap1 structure. G in orange, guanylate in Cap1 structure. αG, human α-Globin. UTR, untranslated region. poly(A)120, a polyadenylate tail of 120 nucleotides long. OVA, chicken ovalbumin. HLA, human leukocyte antigen. SP, signal peptide. FL, full-length. T&CD, transmembrane and cytoplasmic domain. NAP10, 10 neoantigen peptides linked in tandem. EGFP, enhanced green fluorescent protein. S2P, SARS-CoV-2 spike protein with K986P and V987P substitutions. **e**–**h** Particle size, zeta potential, encapsulation efficiency and polydispersity index of OVA cm vaccine and OVA pm vaccine. **i** Transmission electron microscopy images of cm Vaccine and pm Vaccine. Scale bar on the lower left corner of each snapshot, 100 nm. **j** In vivo biodistribution of cm Vaccine and pm Vaccine visualized by luciferase expression 6 h after intramuscular injection

Pam2Cys and its derivatives signal through TLR2/6 pathway, have been shown to elicit both humoral and cellular adaptive immune responses.^17–24^ An inhalable drug, PUL-042, containing a Pam2Cys derivative, has been tested for efficacy against bacterial and viral infections.^25^ Pam2Cys is a simple synthetic neutral lipoamino acid that are easily attainable and metabolizable, consisting of only a cysteine, a thioglycerol and two fatty acid residues (Fig. 1a). And, unlike nucleotide/nucleic acid and other small-molecule adjuvants, its lipophilicity facilitates the incorporation into the lipid layers of lipid-based nanovehicles, without the need of replacing a proportion of mRNA composition or any further chemical modification to achieve co-delivery with the antigen-encoding mRNA payload. Last but not the least, Pam2Cys is of high potency, half maximally active at 10^−11 ^M concentration.^26^ Thus, Pam2Cys is an interesting candidate adjuvant in these regards. To confirm that Pam2Cys improves the efficacies of mRNA vaccines, the adjuvant was incorporated in mRNA-LNPs and the resulting new vaccine formulations were put to test in cancer models and a COVID-19 surrogate model.

Previous studies focused on investigation of Pam2Cys-conjugated peptide epitopes as vaccines or its water-soluble derivative Pam2CSK4 as a drug in aqueous solution without any vaccine. In this study, by exploiting its adjuvanticity and hydrophobicity, Pam2Cys was incorporated into mRNA-LNPs in order to boost the efficacy of mRNA vaccines. The potency of this new vaccine formulation was tested in several disease models to provide data for potential future clinical application.

## Results

### Preparation of Pam2Cys-incorporated mRNA vaccines

Pam2Cys (Fig. 1a), at the molar ratio of 0.5%, was used alongside the four lipids that make the conventional LNPs^27^ to encapsulate mRNA (Fig. 1b–d). The resulting Pam2Cys-incorporated mRNA-LNPs, hereafter termed pm vaccine, were smaller in size (82.690 ± 2.394 versus 89.073 ± 1.679 nm) (Fig. 1e), than conventional mRNA-LNPs, hereafter termed cm vaccine. However, the two vaccines were similar in zeta potential (−4.184 ± 0.832 versus −3.519 ± 0.914 mV) (Fig. 1f), encapsulation efficiency (90.897 ± 0.694% versus 89.50 ± 0.540%) (Fig. 1g) and they both exhibited decent polydispersity indexes (0.110 ± 0.007 versus 0.128 ± 0.011) (Fig. 1h). Imaging by transmission electron microscopy revealed that the two vaccines were both suspensions of generally oval-shaped nanoparticles (Fig. 1i). Injection of the two vaccines into the hind thigh muscle both generated similar level of target gene expression at the vaccination site, and, to a lesser extent, in the liver (Fig. 1j).

### Shaping of the immune milieu and antigen presentation by pm vaccine in the dLNs

**Fig. 2:**
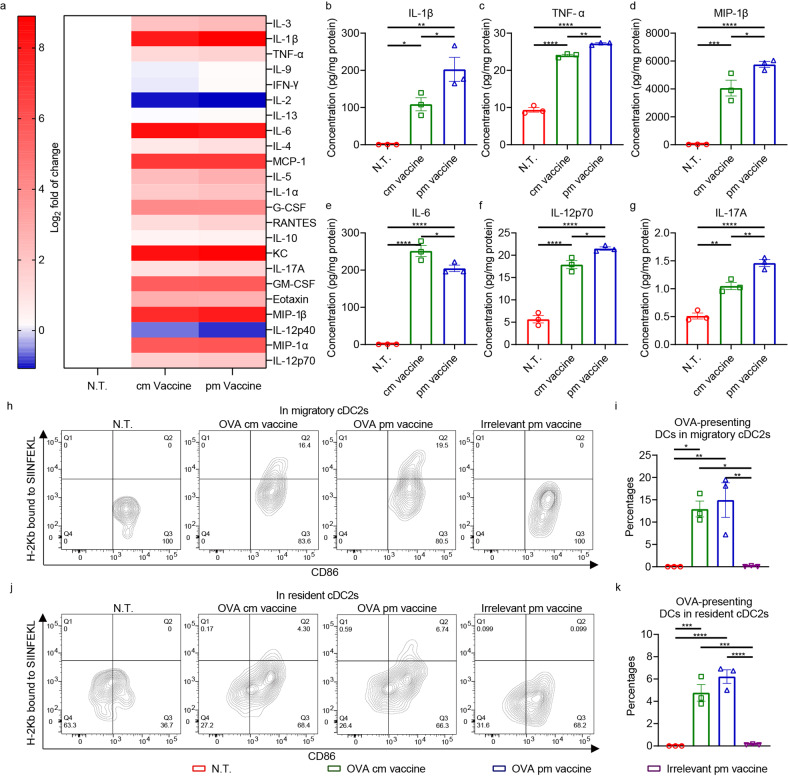
Shaping of the immune milieu and antigen presentation by pm vaccine in the dLNs. **a**–**g** Cytokine concentrations in the dLNs 24 h after immunization of C57BL/6 mice with an i.m. dose of mRNA vaccines containing 20 μg OVA mRNA were measured with Luminex multiplex cytokine assay (*n* = 3 per group). **a** Heatmap displaying changes in the cytokine levels. Log_2_ fold of changes of means relative to that of non-treated (N.T.) controls are plotted in the heatmap. **b**–**g** Bar charts of cytokine concentrations that was significantly altered. **h**, **i** Flow cytometric analysis of MHC-I antigen presentation (H-2Kb bound to OVA epitope SIINFEKL) in migratory cDC2s in dLNs 24 h after immunization of C57BL/6 mice with an i.m. dose of mRNA-LNPs containing 20 μg OVA mRNA. **j**, **k** Flow cytometric analysis of MHC-I antigen presentation in resident cDC2s in dLNs 24 h after immunization of C57BL/6 mice with an i.m. dose of mRNA-LNPs containing 20 μg OVA mRNA (*n* = 3 per group). Data are shown as means with SEM. Statistical significances were determined by one-way ANOVA with Dunnett’s post hoc tests. *P < 0.05. **P < 0.01. ***P < 0.001. ****P < 0.0001

OVA pm vaccine led to prominent changes in cytokine levels in the dLNs 24 h after intramuscular (i.m.) administration (Fig. 2a), showing significantly elevated level of proinflammatory cytokines (IL-1β and TNF-α) and chemokine MIP-1β, compared to OVA cm vaccine (Fig. 2b–d). Counterintuitively, the level of one other proinflammatory cytokine, IL-6, was significantly reduced (Fig. 2e). Interestingly, the levels of Th1 cytokine IL-12 p70 and Th17 cytokine IL-17A, rather than that of Th2 cytokine IL-4, were significantly heightened (Fig. 2f, g, Supplementary Fig. 1a), suggesting the induction of Th1- and Th17-biased immune responses. There were no significant differences between cm and pm vaccine groups in levels of other cytokine detected, including other chemotactic ones, such as MIP-1α, RANTES, and MCP-1 (supplementary Fig. 1b–q).

Flow cytometric analysis of cells in dLNs revealed that OVA-derived CD8^+^ T cell epitope (SIINFEKL) was presented by migratory cDC2s through major histocompatibility complex class I (MHC-I) pathway, and, to a lesser extent, by dLN-resident cDC2s (Fig. 2h, i),^28–30^ which indicated efficient expression and processing of mRNA-encoded antigen. The presence of OVA epitope-presenting cDC2s in pm vaccine group was of higher frequency than in cm vaccine group, albeit not statistically significant. Moreover, the DCs presenting the epitope were in active state, as reflected by their CD86 upregulation (Fig. 2j, k). Surprisingly, antigen presentation by both migratory and dLN-resident cDC1s was of low frequency (supplementary Fig. 3).

cDC2s were therefore checked after vaccination for secretion of the forementioned cytokines, IL-12 p70 and IL-6, specifically. Flow cytometry analysis revealed that elevated proportion of cDC2s in the dLNs secreted IL-12 p70 after vaccination, with migratory cDC2s much more prominent than resident ones (supplementary Fig. 4a–d). Vaccination also induced IL-6 secretion in cDC2s, in lower proportion in pm vaccine group than that in cm vaccine group (supplementary Fig. 4e–h). These facts suggested that cDC2s also contributed to the secretion of important cytokines that shaped the immune milieu in the dLNs.

These results demonstrated that immunization with pm vaccine led to shaping of immune responses through the induction of IL-12 and IL-17, among other cytokines, and efficient antigen presentation to T cells by both migratory and resident cDC2s in the draining lymph nodes.

### Robust T cell-mediated inhibition of established E.G7-OVA tumors and generation of immune memory by OVA pm vaccine

**Fig. 3:**
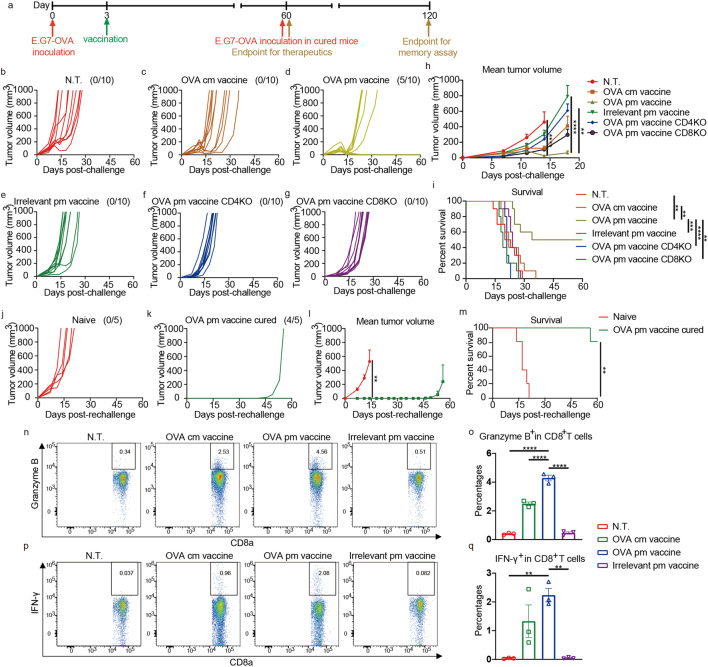
Robust T cell-mediated inhibition of established E.G7-OVA tumors and generation of immune memory by OVA pm vaccine. **a** Wild-type, as well as CD4-KO and CD8a-KO C57BL/6 mice were challenged s.c. on day 0 with 3 × 10^5^ E.G7-OVA cells, then immunized c.l. on day 3 with an i.m. dose of mRNA vaccines containing 20 μg OVA mRNA (*n* = 10 per group). Mice cured of the initial E.G7-OVA tumors were rechallenged with 3 × 10^5^ E.G7-OVA cells on day 60 to assay for memory responses (*n* = 5 per group). **b**–**g** Individual growth curves of E.G7-OVA tumors in mice in therapeutic assay. Fractions in the parentheses indicate the proportion of cured mice in the groups. **h** Mean volumes of E.G7-OVA tumors in mice in therapeutic assay. Data are shown as means with SEM. Statistical significances were determined by one-way ANOVA with Dunnett’s post hoc tests. **i** Survival curves of mice bearing E.G7-OVA tumors in therapeutic assay. Statistical significances were determined by log-rank test. **j**, **k** Individual growth curves of E.G7-OVA tumors in mice in memory assay. Fractions in the parentheses indicate the proportion of tumor-free mice in the groups. **l** Mean volumes of E.G7-OVA tumors in mice in memory assay. Data are shown as means with SEM. Statistical significance was determined by unpaired student’s t test. **m** Survival curves of mice challenged with E.G7-OVA tumors in memory assay. Statistical significance was determined by log-rank test. **n**–**q** Splenocytes from mice 70 days after single vaccination (*n* = 3 per group) were restimulated by OVA T cell epitopes and stained for intracellular proteins. **n**, **o** Ratios of Granzyme B^+^ cells in CD8^+^ T cells. **p**, **q** Ratios of IFN-γ^+^ cells in CD8^+^ T cells. Data are shown as means with SEM. Statistical significances were determined by one-way ANOVA with Dunnett’s post hoc tests. **P < 0.01. ***P < 0.001. ****P < 0.0001

The efficacy of the new-model mRNA vaccine was first tested on E.G7-OVA tumor model in a therapeutic setting, with OVA as the target antigen. Mice were inoculated with 3 × 10^5^ E.G7-OVA cells subcutaneously (s.c.). Then a single i.m. dose of pm vaccine containing 20 μg OVA mRNA was given on the contralateral (c.l.) side three days later (Fig. 3a). The regimen eradicated the established tumors in 50% of the mice in OVA pm vaccine group, which was in sharp contrast to 0% in OVA cm vaccine group and other groups (Fig. 3b–e). Immunization with OVA pm vaccine significantly ameliorated tumor burdens and registered significantly improved survival (Fig. 3h, i). Similar efficacies were also documented when administrating via subcutaneous and intravenous routes (supplementary Fig. 5).

The efficacy of OVA pm vaccine was almost completely abrogated in both CD4 and CD8a gene knockout (KO) mice, suggesting a heavy dependence on both CD4^+^ and CD8^+^ T cell immunity in combat with tumor (Fig. 3f–i).

The mice cured of E.G7-OVA tumors with OVA pm vaccine immunization were rechallenged 60 days after the initial tumor inoculation. 80% of the mice rejected the new inocula, and the only one that eventually beared tumor showed significantly postponed emergence of tumor (Fig. 3j, k). Mean tumor burden was sharply reduced and survival was significantly improved (Fig. 3l, m).

OVA-specific memory T cells were detected by flow cytometric analysis of peptide-restimulated splenocytes 70 days after a single immunization. Significantly higher proportion of CD8^+^ T cells secreted Granzyme B (Fig. 3n, o), and also higher proportion of CD8^+^ T cells secreted IFN-γ (Fig. 3p, q) in OVA pm vaccine group than that in OVA cm vaccine group, suggesting more sustainable immune memory were established in OVA pm vaccine group.

These results suggested that immunization with OVA pm vaccine elicited CD4^+^ and CD8^+^ T cell immune responses that efficiently inhibited E.G7-OVA tumors and generated robust immune memory.

### Strengthened prevention of E.G7-OVA tumors by OVA pm vaccine in the prophylactic setting

**Fig. 4:**
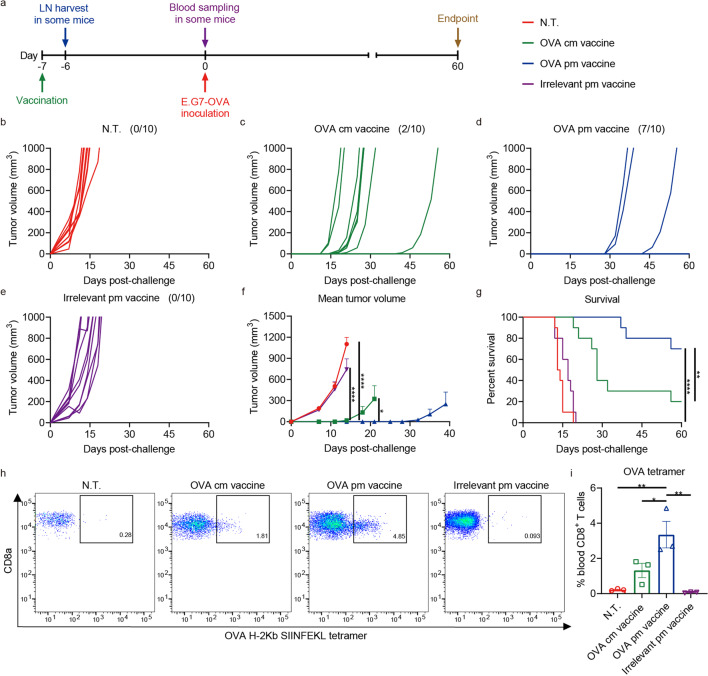
Strengthened prevention of E.G7-OVA tumors by OVA pm vaccine in the prophylactic setting. **a** C57BL/6 mice were immunized on day −7 with an i.m. dose of mRNA vaccines containing 20 μg OVA mRNA and challenged c.l. on day 0 with 5 × 10^5^ E.G7-OVA cells. Some mice were euthanized on day −6 to harvest dLNs for analysis depicted in Fig. 2. Blood samples were collected from some mice for tetramer analysis. **b**–**e** Individual growth curves of E.G7-OVA tumors in mice in prophylactic assay (*n* = 10 per group). Fractions in the parentheses indicate the proportion of tumor-free mice in the groups. **f** Mean volumes of E.G7-OVA tumors in mice in prophylactic assay. Data are shown as means with SEM. Statistical significances were determined by one-way ANOVA with Dunnett’s *post hoc* tests. **g** Survival curves of mice challenged with E.G7-OVA tumors in prophylactic assay. Statistical significances were determined by log-rank test. **h**, **i** Flow cytometric analysis of induced circulating OVA-specific CD8^+^ T cells on day 0 (*n* = 3 per group). H-2Kb SIINFEKL tetramer was used for the analysis. Data are shown as means with SEM. Statistical significances were determined by one-way ANOVA with Dunnett’s post hoc tests. *P < 0.05. **P < 0.01. ****P < 0.0001

The efficacy of OVA pm vaccine was then evaluated in a prophylactic setting. Mice were immunized with a single i.m. dose of pm vaccine containing 20 μg OVA mRNA, and challenged 7 days later c.l. with 5 × 10^5^ E.G7-OVA cells (Fig. 4a). 70% of the mice in OVA pm vaccine group stayed tumor-free, while only 20% in OVA cm vaccine group managed to do so (Fig. 4b–e). Tumor emergences were significantly delayed, burdens were prominently reduced, and survival was greatly improved in OVA pm vaccine group, compared to other groups (Fig. 4f, g).

Flow cytometric analysis revealed a significantly higher ratio of OVA-specific CD8^+^ T cells in circulation 7 days after immunization in OVA pm vaccine group (Fig. 4h, i), suggesting efficient activation and mobilization of antigen-specific T cells.

These results indicated that immunization with OVA pm vaccine efficiently protected mice from E.G7-OVA tumors in a prophylactic setting and generated reinforced OVA-specific T cell immunity.

### Enhanced inhibition of established CT26 tumors by CT26 neoantigen pm vaccine

The efficacy of pm vaccine was further assessed on CT26 model, using 10 tandem linked neoantigen epitopes as target antigens. These mutation-generated epitopes (supplmentary Table S1) were selected from those immunogenic ones mined from CT26 tumor cells.^31^

**Fig. 5:**
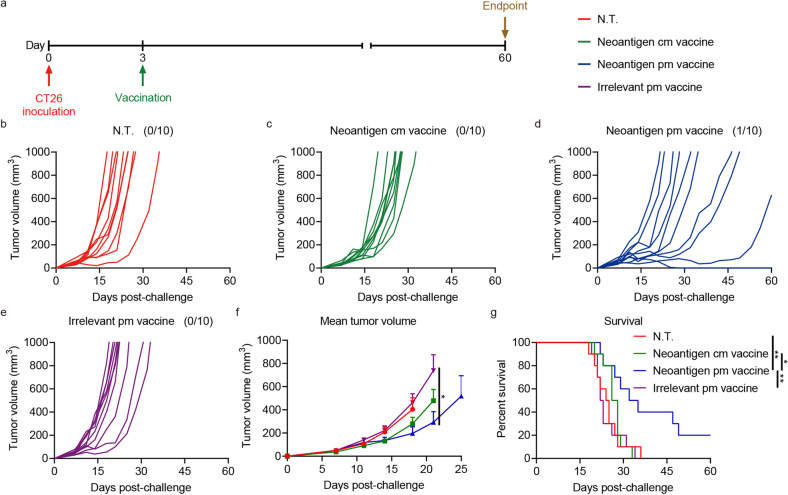
Enhanced inhibition of established CT26 tumors by CT26 neoantigen pm vaccine. **a** Balb/c mice were challenged s.c. on day 0 with 3 × 10^5^ CT26 cells, then immunized c.l. on day 3 with an i.m. dose of mRNA vaccines containing 20 μg CT26 neoantigen mRNA (*n* = 10 per group). **b**–**e** Individual growth curves of CT26 tumors in mice in therapeutic assay. Fractions in the parentheses indicate the proportion of cured mice in the groups. **f** Mean volumes of CT26 tumors in mice in therapeutic assay. Data are shown as means with SEM. Statistical significances were determined by one-way ANOVA with Dunnett’s post hoc tests. **g** Survival curves of mice bearing CT26 tumors in therapeutic assay. Statistical significances were determined by log-rank test. *P < 0.05. **P < 0.01

Mice were inoculated s.c. with 3 × 10^5^ CT26 cells, and i.m. immunized with a single dose of pm vaccine containing 20 μg CT26 neoantigen mRNA on the contralateral side three days later (Fig. 5a). The regimen eradicated the established tumor in 10% of the mice in CT26 neoantigen pm vaccine group, retarded tumor growth, and registered significantly improved survival (Fig. 5b–g).

These results demonstrated that the pm vaccine was also efficacious on the CT26 tumor therapeutic model and held the promise of general applicability.

### Augmented humoral and cellular adaptive immune responses triggered by SARS-CoV-2 spike pm vaccine

After the elucidation of potent activation of cellular immunity by pm vaccine, it was also intriguing to see the effects of pm vaccine on the humoral arm of the adaptive immunity. COVID-19 vaccines have been being under heated research and development since the outbreak of the pandemic, and was a proper model to test the humoral responses triggered by pm vaccine.

**Fig. 6:**
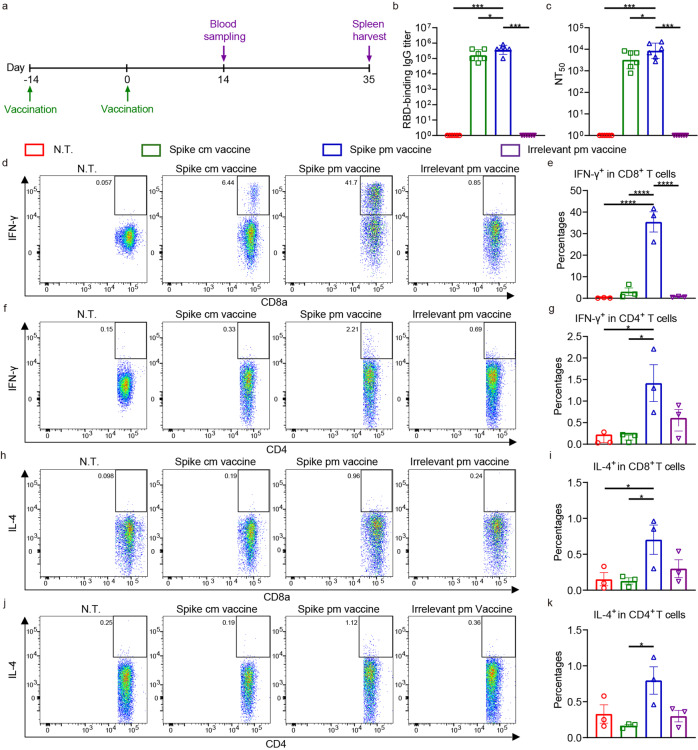
Augmented humoral and cellular adaptive immune responses triggered by SARS-CoV-2 spike pm vaccine. **a** C57BL/6 mice were primed on day −14 with an i.m. dose of mRNA vaccines containing 20 μg spike mRNA and boosted c.l. likewise on day 0. Blood samples were collected on day 14 for antibody titer measurement (*n* = 6 per group). Spleens were harvested on day 35 for intracellular cytokine staining (*n* = 3 per group). **b** Measurement of RBD-binding IgG titers in peripheral blood. **c** Measurement of neutralization antibody titers in peripheral blood against spike pseudoviruses. NT_50_, neutralizing antibodies with 50% neutralization titer. Data are shown as geometric means with SD. **d**–**k** Splenocytes from immunized mice were restimulated by peptide pool spanning SARS-CoV-2 spike protein and stained for intracellular cytokines. **d**, **e** Ratios of IFN-γ^+^ cells in CD8^+^ T cells. **f**, **g** Ratios of IFN-γ^+^ cells in CD4^+^ T cells. **h**, **i** Ratios of IL-4^+^ cells in CD8^+^ T cells. **j**, **k** Ratios of IL-4^+^ cells in CD4^+^ T cells. Data are shown as means with SEM. Statistical significances were determined by one-way ANOVA with Dunnett’s post hoc tests. *P < 0.05. ***P < 0.001. ****P < 0.0001

Mice were i.m. primed with a dose of pm vaccine containing 20 μg SARS-CoV-2 spike mRNA, and boosted likewise 14 days later (Fig. 6a). After another 14 days, peripheral blood sera were collected, and assayed for specific antibody titers. Spike pm vaccine triggered significantly higher receptor-binding domain (RBD)-binding IgG titers (Fig. 6b) and spike pseudovirus-neutralizing antibody titers (Fig. 6c), compared to that of Spike cm vaccine.

Beside humoral immunity, vaccine-induced T cell immunity was also evaluated 35 days after the last vaccination. Intracellular cytokine staining of splenocytes incubated with peptide pool of SARS-CoV-2 spike protein were performed to assess subsets of restimulated T cells. Drastically more IFN-γ^+^CD8^+^ T cells were detected in Spike pm vaccine group, compared to that in any other group (Fig. 6d, e). These cells expressed somewhat higher levels of IFN-γ, compared to that of the Spike cm vaccine group (supplementary Fig. 6a). Furthermore, there was a significantly higher presence of IFN-γ^+^ cells in CD4^+^ T cells in Spike pm vaccine group (Fig. 6f, g). These cells expressed significantly higher levels of IFN-γ, compared to that in any other group (supplementary Fig. 6b). In addition, significantly more IL-4^+^CD8^+^ T cells and IL-4^+^CD4^+^ T cells were also detected in Spike pm vaccine group, compared to that in Spike cm vaccine group (Fig. 6h, k). These facts demonstrated Spike pm vaccine potently activated antigen-specific cytotoxic T lymphocytes, Th1-polarized CD4^+^ T and Th2-polarized CD4^+^ T.

These Data indicated that immunization with SARS-CoV-2 spike pm vaccine stimulated strong antigen-specific humoral and cellular adaptive immune responses, which might offer immunoprotection against the pathogen.

### Good preliminary safety profile of pm vaccine in mice

**Fig. 7:**
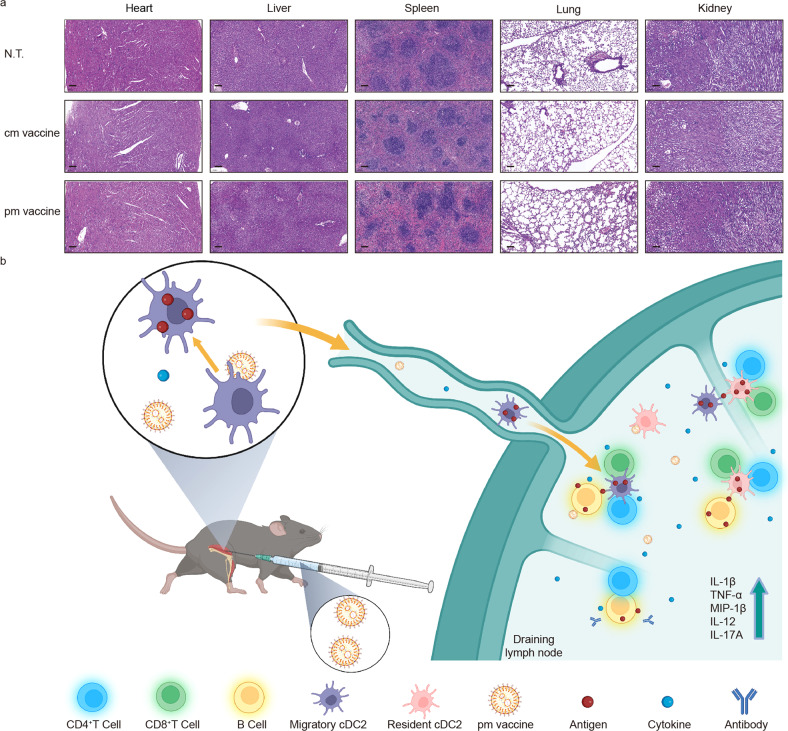
Good preliminary safety profile of pm vaccine. **a** Histological sections of vital organs of immunized mice 45 days after the last vaccination. Scale bar on the lower left corner of each snapshot, 100 μm. **b** Mechanism of action for Pam2Cys-incorporated mRNA vaccines. This schematic illustration was created by Biorender

Mice administrated with pm vaccine exhibited no alterations on gross features, such as weight loss, ruffling of fur, behavior, etc., during the 70-day follow-up. Meanwhile, there were no histopathological changes in vital organs, e.g., heart, liver, spleen, lung and kidney (Fig. 7a) in euthanized mice 45 days after pm vaccine immunization.

These preliminary data suggested that pm vaccine exhibited a good safety profile in mice.

## Discussion

In this study, we have shown that immunization of mRNA vaccines incorporated with Pam2Cys shaped up the immune responses in draining lymph nodes through the induction of IL-12 and IL-17, among other cytokines. Antigen presentation was carried out mainly by cDC2s of migratory and dLN-resident DCs and potent antitumor responses were generated in both prophylactic and therapeutic tumor models. These immune responses were highly depended on both CD4^+^ and CD8^+^ T cells. Memory antitumor immunity was also established by this new mRNA vaccine formulation. Moreover, the vaccine also reinforced humoral and cellular immunity in a surrogate COVID-19 prophylactic model. Last but not the least, preliminary data showed that the vaccines were safe in murine models.

There is a generally believed dichotomous concept that cDC1s are adept at cross-presentation and CD8^+^ T cell activation, while cDC2s are specialized in CD4^+^ T cell activation.^32^ However, several recent discoveries challenged this paradigm. It was proposed that cDC2s could also efficiently prime CD8^+^ T cells in addition to CD4^+^ T cells.^33–36^ Our findings that CD8^+^ T cells were prominently induced in different models were consistent with their results. This CD8^+^ T cell priming was dependent on type I interferon signals,^35,36^ which could be offered by mRNA vaccines^37^ and amplified by signaling mediated by Pam2Cys family molecules^38^ in our case. Therefore, our data were in agreement with these new findings. The level of IL-6 was significantly reduced in dLNs of pm vaccine immunized mice, compared to that of cm vaccine immunized ones, suggesting an inhibition of excessive inflammation in pm vaccine group.^39,40^ And since the previously less appreciated immunosuppressive roles of IL-6 have been unveiled in recent years,^41–44^ this IL-6 downregulation might be beneficial to antitumor immunity. IL-17A was significantly upregulated in dLNs after immunization with pm vaccine compared to cm vaccine. Unlike IL-12, IL-17 plays much more controversial roles in immunity. For example, it can be either tumor-promoting and tumor-inhibiting, in a context-dependent manner.^45–47^ The antitumor effects of IL-17 are mediated by T cells.^48^ IL-17 can drive Th1 immune responses by overcoming IL-10-mediated inhibition^49^ and inducing IL-12 production in DCs.^50–52^ In addition to cellular immunity, IL-17 also has great influence on humoral immunity. IL-17 promotes the formation of germinal centers,^53,54^ where plasma cells and memory B cells that secret high-affinity antibodies against antigens are fostered. Thus, IL-17 might be an important participant in augmenting the pm vaccine-induced immune responses in our scenarios. Noteworthily, lower proportion of cDC2s from pm vaccine group secreted IL-12 p70 than that from cm vaccine group, which was in contrast with the fact that IL-12 p70 levels in the dLNs from pm vaccine group were higher than that from cm vaccine group. The inconsistency might suggest that there were other cellular sources of IL-12 p70, and/or different levels of IL-12 p70 originating from vaccination sites trafficked to the dLNs.

Contrary to our and numerous other reports advocating the adjuvant effects on immune responses, there were studies suggesting an immunosuppressive role of Pam2Cys family by promoting the activity of monocytic myeloid-derived suppressor cells. However, this negative influence can be abrogated by insulation of T cells from these suppressor cells.^55,56^ In these studies, water-soluble Pam2CSK4 was administrated intraperitoneally, intravenously or subcutaneously around tumors. It would be interesting to elucidate to what extent the proposed immunosuppressive effects are route- and formulation-dependent in future study, so that potential improvements in the current pm vaccine can be made.

The more frequently used TLR2 agonists in immunological studies, Pam3CSK4^14,57,58^ and Pam2CSK4, are water-soluble, and thus prone to shed from the nanovehicles, which may compromise the stability and efficacy of mRNA vaccines. To circumvent this shortcoming, we opted to use the water-insoluble and lipophilic version, Pam2Cys, which is the simplest in structure in its kind. Furthermore, Pam2Cys is more potent than the TLR1/2 agonist Pam3Cys in activating the immune system.^18,59,60^ Thus, Pam2Cys is the appropriate option in these regards. There is a chiral center in the Pam2Cys molecule, and the commercially available chemical is actually a mixture of *R* and *S* diastereomers. Since the *R* diastereomer proved to be significantly more potent than the *S* one as an immunostimulator,^60^ incorporation of the *R* diastereomer of Pam2Cys into mRNA vaccines might bring about better efficacies. However, on the other hand, this might not be so economically friendly under current situation. Thus, Pam2Cys is the choice TLR2/6 agonist for adjuvanticity in LNP-based mRNA delivery, and the resulting Pam2Cys-incorporated LNPs hold promise for application in future clinical immune-based interventions.

In conclusion, our data demonstrated that incorporation of Pam2Cys potentiated mRNA vaccines against cancer and infectious diseases. The vaccine formulation stimulated both the cellular and humoral arms of the adaptive immune system further beyond what conventional mRNA-LNPs can do. These results indicated that Pam2Cys-incorporated mRNA vaccines are generally applicable for improving mRNA vaccine performances in future clinical practices.

## Materials and methods

### Reagents, cell lines, and animals

Cholesterol, DSPC (also known as 1,2-Distearoyl-sn-glycero-3-phosphorylcholine) and DMG-PEG2000 (also known as 1,2-Dimyristoyl-sn-glycero-3-methoxypolyethylene glycol 2000) were purchased from AVT (Shanghai, China). ALC-0315 (also known as (4-hydroxybutyl) azanediyl) bis (hexane-6,1-diyl) bis (2-hexyldecanoate), was purchased from SINOPEG (Xiamen, China). Pam2Cys (also known as S-[2,3-bis (Palmitoyloxy) Propyl]-L-Cysteine), was purchased from TGpeptide (Nanjing, China).

Murine colon carcinoma CT26 cells were maintained in RPMI 1640 medium supplemented with 10% fetal bovine serum (Thermo Fisher Scientific, Waltham, MA, USA). Murine lymphoma E.G7-OVA cells were maintained in RPMI 1640 medium supplemented with 10% fetal bovine serum and 400 μg/mL G418 (InvivoGen, San Diego, CA, USA). These cells were obtained from ATCC, and free of mycoplasma contamination.

Female C57BL/6 and Balb/c mice were purchased from HFKbio (Beijing, China) and used at the age of 8–10 weeks. CD4 KO and CD8a KO mice (C57BL/6 genetic background) were purchased from the Jackson Laboratory (Bar Harbor, ME, USA), bred in our facility, and females were used at the age of 8–10 weeks. Animals were allocated to groups with randomization.

### DNA vector construction and mRNA production

For the OVA-encoding DNA template vector, A DNA insert encoding an HLA-A signal peptide, a chicken ovalbumin coding sequence and an HLA-B transmembrane and cytoplasmic domains was cloned between 5’ and 3’ α-globin UTRs into a synthetic plasmid vector pT7ggAGA, which also contains a T7 promoter and a 120nt-poly(A) sequence flanking the UTRs, respectively. For the CT26 neoantigen-encoding DNA template vector, the forementioned coding sequence of chicken ovalbumin was replaced with that of 10 tandem CT26 neoantigen peptides^31^ joined with (GGGGS)×2 linkers. For the irrelevant control EGFP-encoding DNA template vector, the forementioned coding sequence of chicken ovalbumin was replaced with that of EGFP. For the SARS-CoV-2 spike protein-encoding DNA template vector, the full-length spike protein coding sequence from the prototype virus strain with K986P and V987P mutations was cloned between 5’ and 3’ α-globin UTRs into pT7ggAGA.

The vectors were linearized with *Bsp*Q I (Hongene, Shanghai, China) at the very end of poly(A) sequence, and used as templates for in vitro transcription (IVT) by T7 RNA polymerase (Hzymes Biotech, Wuhan, China). UTP was replaced by N1-methyl pseudouridine triphosphate (Glycogene, Wuhan, China) in the IVT reaction. Posttranscription capping was performed with vaccinia capping enzyme and 2’-O-methyltransferase (Hzymes Biotech, Wuhan, China). mRNAs were purified with oligo (dT)30 magnetic beads (Vdobiotech, Suzhou, China), quantified by Nanodrop 2000 Spectrophotometer (Thermo Fisher Scientific), and assessed for quality by denaturing formaldehyde agarose gel electrophoresis.

### Formulation and characterization of mRNA-LNPs

mRNA in citric buffer (10 mM, pH 3.0) were mixed with lipids in ethanol using iNano microfluidic device (Micro & Nano, Shanghai, China) at the speed of 12 mL/min, the volume ratio of 3 and the nitrogen/phosphate ratio of 6 to form mRNA-LNPs. The lipids were prepared by dissolving ALC-0315, DSPC, cholesterol and DMG-PEG2000 at the molar ratio of 50:10:38.5:1.5, or ALC-0315, DSPC, cholesterol, DMG-PEG2000 and Pam2Cys at the molar ratio of 49.75:9.95:38.3075:1.4925:0.5. The mRNA-LNPs were dialyzed in a MWCO 6–8 kDa Pur-A-Lyzer dialysis tube (Sigma-Aldrich, St. Louis, MO, USA) against >1000 volume of buffer with 20 mM Tris-HCl (pH7.4) and 9% sucrose.

Particle size, polydispersity index (indicator of size heterogeneity) and zeta potential (indicator of colloidal dispersion stability) of mRNA-LNPs were measured with Zetasizer Pro (Malvern Panalytical, Malvern, UK). mRNA encapsulation efficiency was determined by Quant-it RiboGreen RNA Reagent (Thermo Fisher Scientific) following the manufacturer’s instruction. Briefly, mRNA contents of LNP samples with or without lysis by 1% Triton X-100 were quantified (as Q_tritonP_ and Q_tritonN_, respectively) with EnSight Multimode Microplate Reader (PerkinElmer, Waltham, MA, USA) after addition of Quant-it RiboGreen RNA Reagent. The encapsulation efficiencies (EE) were calculated with the formula, EE = (Q_tritonP _− Q_tritonN_)/Q_tritonP_ × 100%.

### In vivo biodistribution of the vaccines

Female C57BL/6 mice were administrated with an i.m. dose of mRNA-LNPs containing 20 µg of Firefly luciferase mRNA. 6 hours later, mice were administrated intraperitoneally with 150 mg/kg luciferin (Aladdin, Shanghai, China). 15 min later, mice were imaged with IVIS Spectrum In Vivo Imaging System (PerkinElmer, Waltham, MA, USA).

### Animal experiments

In the tumor therapeutic setting, mice were challenged s.c. with tumor cells on Day 0. 3 × 10^5^ E.G7-OVA cells were used for C57BL/6 strain (including CD4-KO and CD8a-KO mice), and 3 × 10^5^ CT26 cells for Balb/c strain, respectively. Mice were then immunized with a single i.m. dose of mRNA-LNPs containing 20 μg OVA and CT26 neoantigen mRNA, respectively, c.l. on Day 3 and left without further intervention. Tumor-free mice that survived the initial E.G7-OVA challenge were rechallenged with 3 × 10^5^ E.G7-OVA cells on day 60 to evaluate memory responses.

In the tumor prophylactic setting, C57BL/6 mice were immunized with a single i.m. dose of mRNA-LNPs containing 20 μg OVA mRNA on Day −7, and then challenged c.l. with 5 × 10^5^ E.G7-OVA cells on Day 0. Some mice were euthanized to harvest lymph nodes draining the vaccination sites on Day −6. Peripheral blood was collected on Day 0 for OVA-specific tetramer assay.

Tumors were measured in perpendicular dimensions for length (*L*) and width (*W*), and tumor volumes (*V*) were calculated with the formula, *V* = 0.5 × *L* × *W*^2^. Tumor-bearing mice were euthanized when either of the following criteria was met: (1) Tumor volumes exceeded 1000 mm^3^. (2) Tumors ulcerated or mice became moribund.

In the surrogate COVID-19 prophylactic setting, C57BL/6 mice were immunized with two i.m. doses of mRNA-LNPs containing 20 μg Spike mRNA on Day −14 and Day 0. Peripheral blood was collected on Day 14 for measurement of serum antibody titers.

The experiments were performed in two replicates, and data were collected in a blinded fashion.

### Measurement of cytokines in lymph nodes draining the vaccination sites

24 hours after vaccination, Inguinal lymph nodes draining the vaccinated thighs were harvested and homogenized in Lysis buffer for Luminex assay (Univ-bio, Shanghai, China) with 1× Protease Inhibitor Cocktail (MedChemExpress, Monmouth Junction, NJ, USA). Cytokines in the homogenates were detected with Luminex 200 (Austin, TX, USA) by Univ-bio company.

### Flow cytometry

For detection of OVA presentation by DCs in the draining lymph nodes, the lymph nodes were mashed through 40 μm cell strainers. Cells were initially stained with LIVE/DEAD Fixable Near IR (780) Viability dye (Thermo Fisher Scientific), incubated with anti-CD16/32 antibody (BioLegend, San Diego, CA, USA, Cat #101302), then stained with Spark UV387-conjugated anti-I-A/I-E antibody (BioLegend, Cat #107670), BV421-conjugated anti-XCR1 antibody (BioLegend, Cat #148216), BV785-conjugated anti-CD86 antibody (BioLegend, Cat #105043), FITC-conjugated anti-CD11c antibody (BioLegend, Cat #117306), PE-conjugated anti-H-2Kb bound to SIINFEKL antibody (BioLegend, Cat #141604) and APC-conjugated anti-SIRPa antibody (BioLegend, Cat #144014).

For detection of IL-12 and IL-6 expression in DCs in the draining lymph nodes, the lymph nodes were mashed through 40 μm cell strainers. Cells were cultured in RPMI-1640 medium supplemented with 10% fetal bovine serum, 50 U/mL penicillin, 50 μg/mL streptomycin (Gibco). Brefeldin A and monensin (5 μg/mL each, MedChemExpress) were added to block protein secretion from cells. 6 hours later, cells were initially stained with LIVE/DEAD Fixable Near IR (780) Viability dye (Thermo Fisher Scientific), incubated with anti-CD16/32 antibody (BioLegend, Cat #101302), then stained with BV785-conjugated anti-I-A/I-E antibody (BioLegend, Cat #107645), Spark Red 718-conjugated CD11c antibody (BioLegend, Cat #117372), BV421-conjugated anti-CD11b antibody (BioLegend, Cat #101236), BV510-conjugated CD8a antibody (BioLegend, Cat #100752), PE-conjugated anti-B220 antibody (BioLegend, Cat #103208). Then cells were fixed, permeabilized, and stained with Alexa Fluor 488-conjugated anti-IL-12 p70 antibody (prepared by labeling anti-IL-12 p70 antibody (BioXCell, Lebanon, NH, USA, Cat #BE0233) with Alexa Fluor 488 Conjugation Kit (Abcam, Cambridge, UK)) and APC-conjugated anti-IL-6 antibody (BioLegend, Cat #504507).

For tetramer assay, blood cells were initially stained with LIVE/DEAD Fixable Near IR (780) Viability dye (Thermo Fisher Scientific), incubated with anti-CD16/32 antibody (BioLegend, Cat #101302), then stained with BV421-conjugated anti-CD3e antibody (BioLegend, Cat #155617), FITC-conjugated anti-CD8a antibody (MBL International, Woburn, MA, USA, Cat #K0227-4) and PE-conjugated H-2Kb SIINFEKL tetramers (MBL International, Cat #TS-5001-1C).

For detection of memory T cell responses against OVA antigen, splenocytes were isolated 70 days after vaccination and cultured in RPMI-1640 medium supplemented with 10% fetal bovine serum, 50 U/mL penicillin, 50 μg/mL streptomycin (Gibco). MHC-I and MHC-II-restricted epitopes of OVA (SIINFEKL and ISQAVHAAHAEINEAGR, respectively, 2 μg/mL each peptide) were added to restimulate cells. Brefeldin A and monensin (5 μg/mL each, MedChemExpress) were added to block protein secretion from cells. 6 h later, cells were initially stained with LIVE/DEAD Fixable Near IR (780) Viability dye (Thermo Fisher Scientific), incubated with anti-CD16/32 antibody (BioLegend, Cat #101302), then stained with PE-conjugated anti-CD44 antibody (BioLegend, Cat #103008), Spark Red 718-conjugated anti-CD3 antibody (BioLegend, Cat #100282), BV421-conjugated anti-CD4 antibody (BioLegend, Cat #100438), and BV510-conjugated anti-CD8a (BioLegend, Cat #100752) antibody. Then cells were fixed, permeabilized, and stained with Alexa Fluor 488-conjugated anti-Granzyme B antibody (BioLegend, Cat #396424) and Alexa Fluor 647-conjugated anti-IFN-γ antibody (BioLegend, Cat #505814).

For detection of T cell responses against SARS-CoV-2 Spike antigen, splenocytes were isolated 35 days after the second vaccination and cultured in RPMI-1640 medium supplemented with 10% fetal bovine serum, 1 mM pyruvate, 50 U/mL penicillin, 50 μg/mL streptomycin (Gibco), 50 μM β-mercaptoethanol and 20 U/mL IL-2 (Sigma-Aldrich). Peptide pool spanning SARS-CoV-2 spike protein (2 μg/mL each peptide) was added to restimulate cells. Before staining, brefeldin A (BD, Franklin Lakes, NJ, USA) was added to block cytokine secretion from cells. Cells were initially stained with BV510-conjugated anti-CD44 antibody (BioLegend, Cat #103044), FITC-conjugated anti-CD4 antibody (BioLegend, Cat #100406), PerCP-conjugated anti-CD3e antibody (BD, Cat #553067) and APC-conjugated anti-CD8a (BioLegend, Cat #100712) antibody. Then cells were fixed, permeabilized, and stained with BV421-conjugated anti-IL-4 antibody (BioLegend, Cat #504120) and PE-conjugated anti-IFN-γ antibody (BioLegend, Cat #505808).

The stained cells were analyzed with LSR Fortessa Flow Cytometer (BD) or Novocyte (Agilent, Santa Clara, CA, USA). Data collected were processed using Flowjo software v10.8 (BD).

### Measurement of SARS-CoV-2 RBD-binding serum IgG titers

SARS-CoV-2 RBD-binding serum IgGs were measured by enzyme-linked immunosorbent assay (ELISA). 96-well high binding plates (Thermo Scientific) were coated with 100 μL of 0.1 μg/mL prototype RBD solution per well for 12 h at 4 °C. Plates were washed and blocked with 1% bovine serum albumin (BSA) for 1 h at 37 °C. Then, 100 μL of serially diluted sera were added to the plates and incubated for 1 h at 37 °C. Following washing steps, the plates were incubated with horseradish peroxidase-conjugated anti-mouse IgG antibody ((Thermo Fisher Scientific) for 1 h at 37 °C. After washing, color development was initiated with 3,3’,5,5’-tetramethylbiphenyldiamine and stopped with 1 M H_2_SO_4_. The absorbance was measured at 450 nm with Spectramax ABS (Molecular Devices, San Jose, CA, USA).

### Measurement of spike pseudovirus neutralization titers

The spike pseudovirus neutralization assay was described previously.^61,62^ Briefly, immune sera were serially diluted and pre-incubated with luciferase-expressing pseudovirus displaying prototype spike protein (Genomeditech, Shanghai, China) in 96-well plates for 1 h at 37 °C. Then 293T/ACE2 cells were seeded at a density of 1 × 10^4^ cells per well and incubated for 48 h. Cells were lysed and luciferase substrates were added following the manufacturer’s (Promega Madison, WI, USA) instructions and relative light units were measured with EnSight Multimode Microplate Reader (PerkinElmer). NT_50_ was calculated by non-linear regression (inhibitor versus normalized response) with GraphPad Prism 8 (San Diego, CA, USA).

### Histopathological study

Hematoxylin and eosin-stained paraffin sections of vital organs (heart, liver, spleen, lung, and kidney) from mice euthanized 45 days after vaccination were prepared and studied for possible histopathological changes.

### Statistical analysis

Data were analyzed with GraphPad Prism 8. Statistical significances were determined by log-rank test, unpaired one-tailed student’s t test and one-way ANOVA with Dunnett’s post hoc tests. Results were presented as means with SEM or geometric means with SD. Comparisons with *P* < 0.05 were considered as statistically significant.

## Supplementary information

SUPPLEMENTAL MATERIAL

## Data Availability

All raw data are available from the corresponding author on reasonable request.
