# Supplementary material for: Incorporation of a Toll-like receptor 2/6 agonist potentiates mRNA vaccines against cancer and infectious diseases
Source: Signal Transduct Target Ther. 2023 Jul 17;8:273. doi: 10.1038/s41392-023-01479-4 (PMC10350459; doi:10.1038/s41392-023-01479-4)
Supplement: Supplementary file 1 — SUPPLEMENTAL MATERIAL [file 41392_2023_1479_MOESM1_ESM.docx]

Supplementary Materials for

**Incorporation of a Toll-like receptor 2/6 agonist potentiates mRNA vaccines against cancer and infectious diseases**

Yangzhuo Gu^1,2^, Jingyun Yang^1^, Cai He^1^, Tingmei Zhao^1^, Ran Lu^3^, Jian Liu^1^, Xianming Mo^3^, Fuqiang Wen^2^, Huashan Shi^1,^

Correspondence to Yangzhuo Gu (email: [yangzhuo_gu@163.com](mailto:yangzhuo_gu@163.com)) or Huashan Shi (email: [shihuashan@scu.edu.cn](mailto:shihuashan@scu.edu.cn)).

**This file includes:**

Figures S1 to S6

Table S1

**
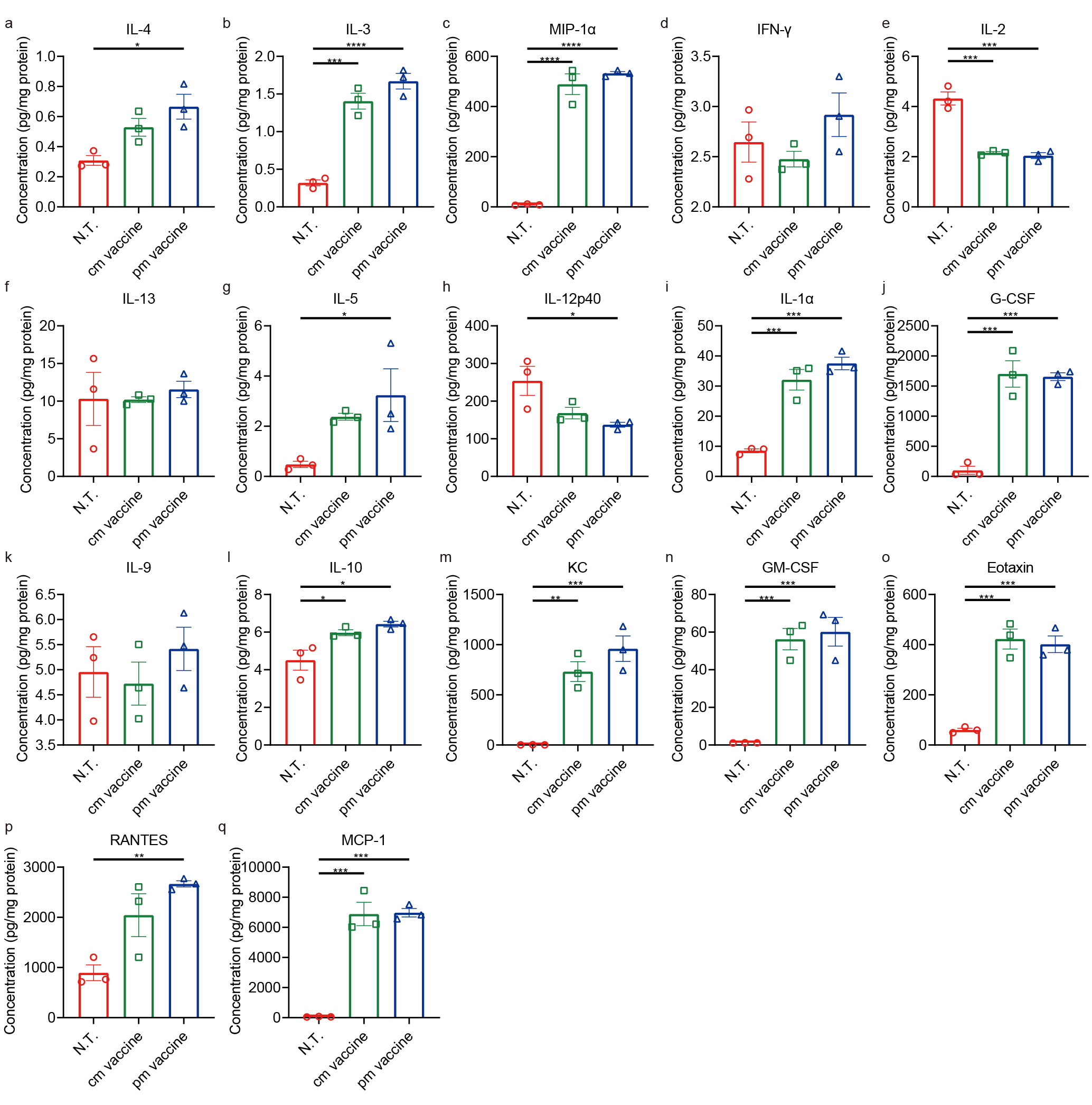
**

**Figure S1. Concentrations of other cytokines in the dLNs 24 hours after immunization.** Mice were immunized with an i.m. dose of mRNA vaccines containing 20 μg OVA mRNA. Cytokine concentrations were measured with Luminex multiplex cytokine assay (n = 3 per group). N.T., non-treated. Data are shown as means with SEM. Statistical significances were determined by one-way ANOVA with Dunnett’s *post hoc* tests. *, P < 0.05. **, P < 0.01. ***, P < 0.001. ****, P < 0.0001.

**
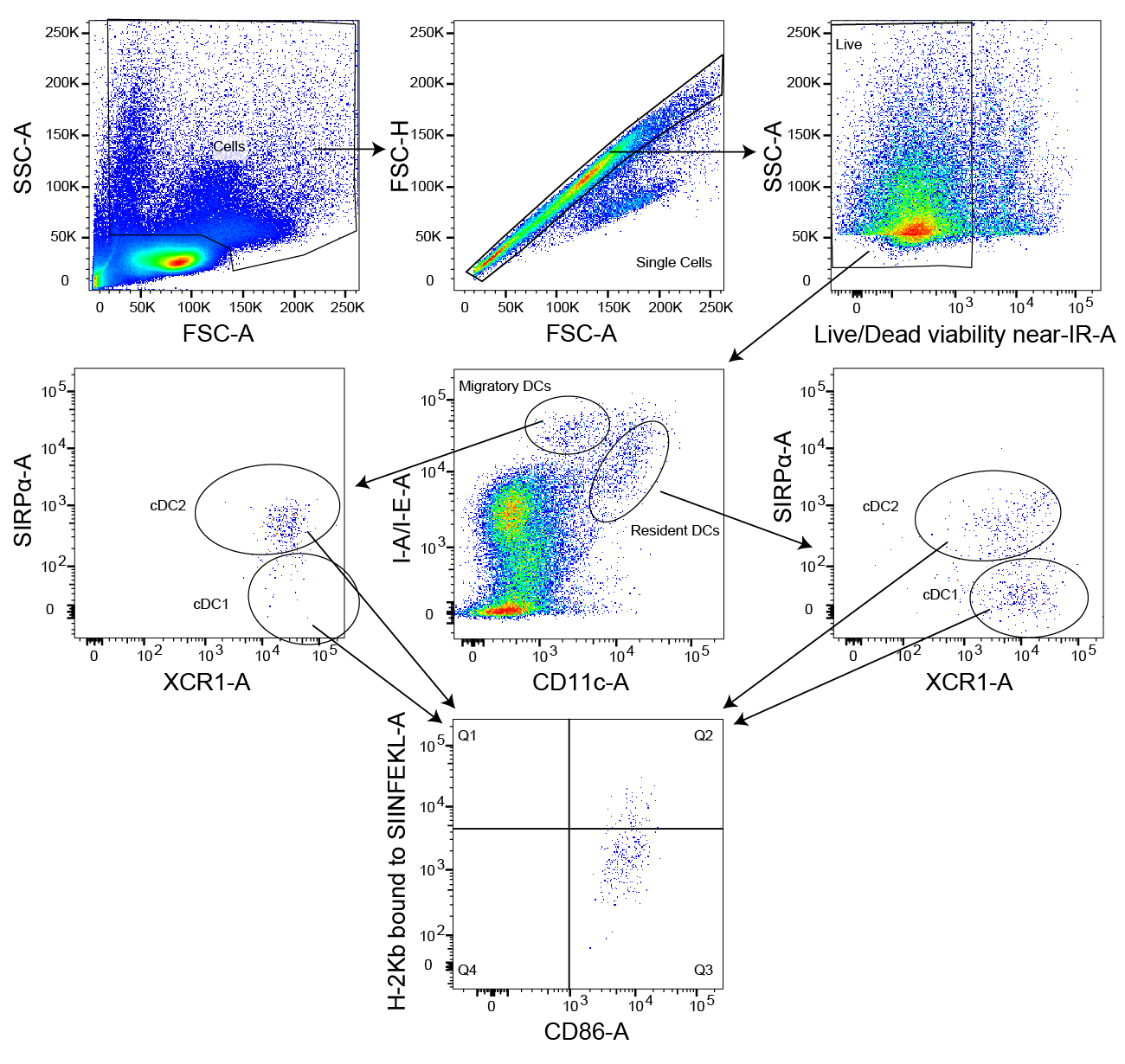
**

**Figure S2. Gating strategy for flow cytometric analysis of MHC-I antigen presentation by DCs in dLNs.** Flow cytometric analysis of MHC-I antigen presentation (H-2Kb bound to OVA epitope SIINFEKL) in DCs in dLNs 24 hours after immunization of C57BL/6 mice with an i.m. dose of mRNA-LNPs containing 20 μg OVA mRNA.

**
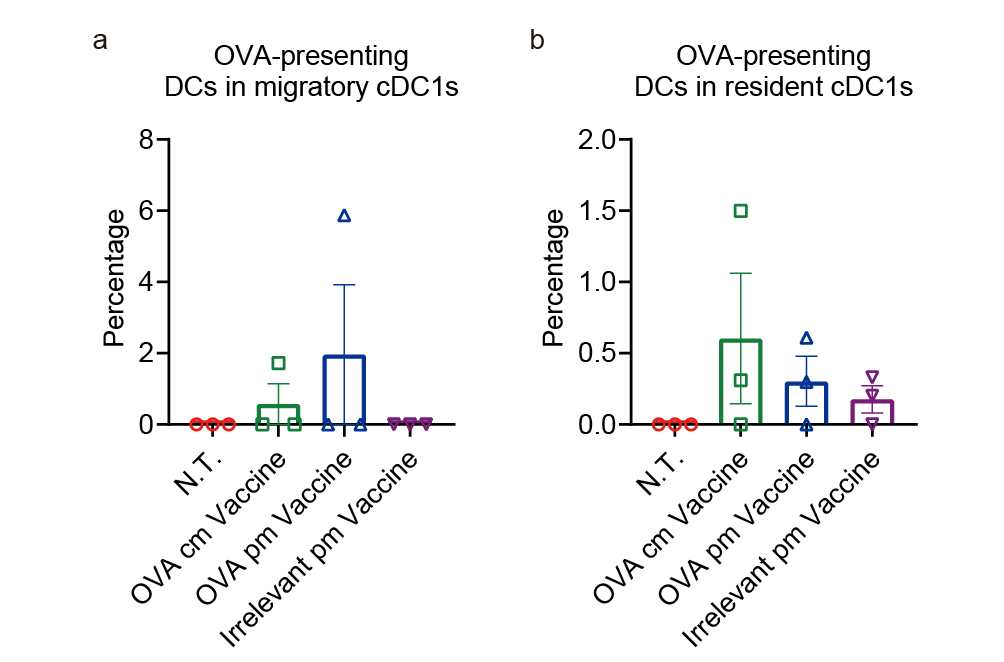
**

**Figure S3. Flow cytometric analysis of MHC-I antigen presentation in cDC1s in dLNs 24 hours after immunization.** (a) Flow cytometric analysis of MHC-I antigen presentation in migratory cDC1s in dLNs 24 hours after immunization of C57BL/6 mice with an i.m. dose of mRNA-LNPs containing 20 μg OVA mRNA. (b) Flow cytometric analysis of MHC-I antigen presentation in resident cDC1s in dLNs 24 hours after immunization of C57BL/6 mice with an i.m. dose of mRNA-LNPs containing 20 μg OVA mRNA (n = 3 per group). N.T., non-treated. Data are shown as means with SEM. Statistical significances were determined by one-way ANOVA with Dunnett’s *post hoc* tests. No significant differences were found among these groups.

**
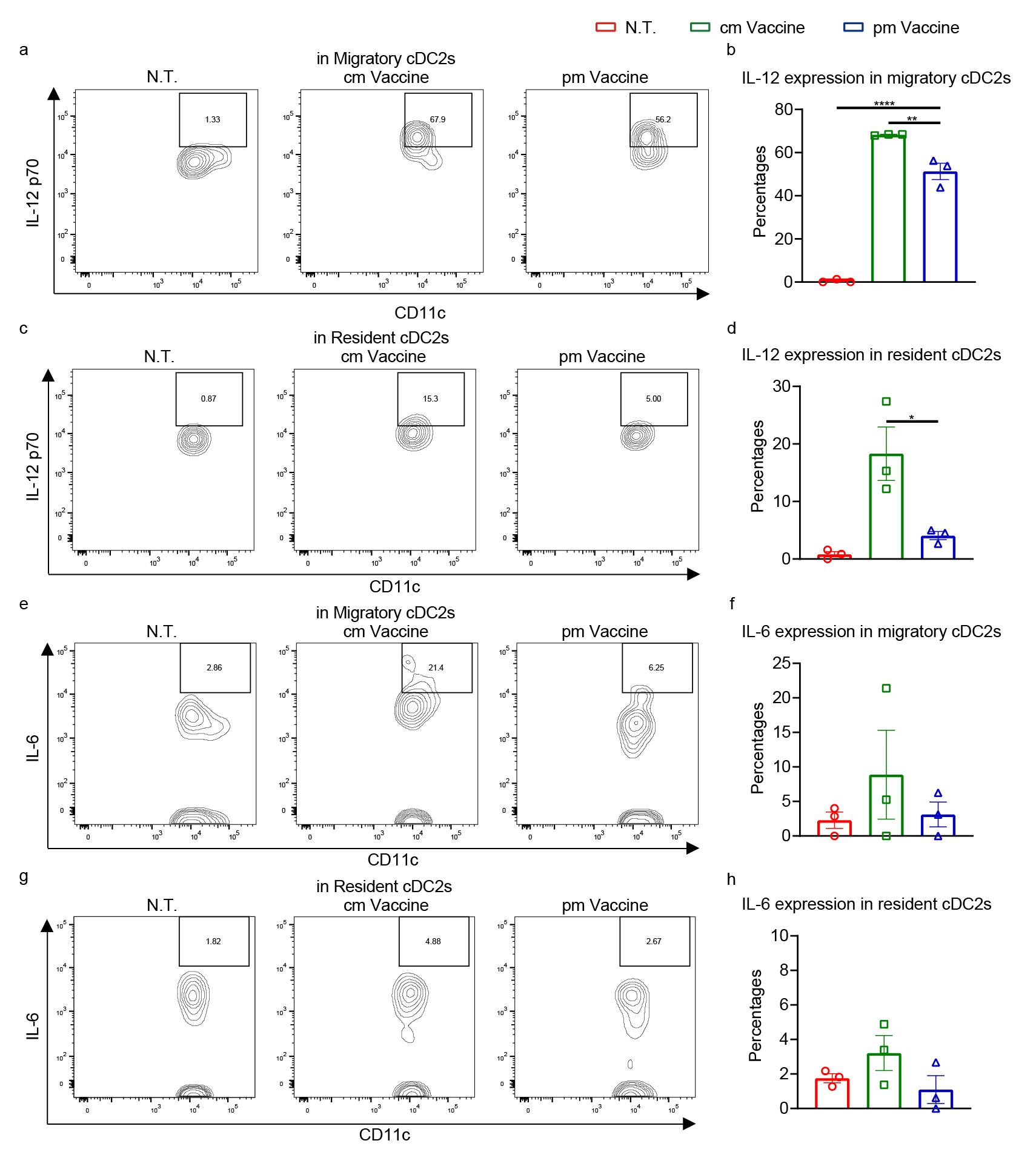
**

**Figure S4. Flow cytometric analysis of IL-12 p70 and IL-6 secretion in cDC2s in dLNs 24 hours after immunization.** Splenocytes were harvested 24 hours after immunization of C57BL/6 mice with an i.m. dose of mRNA-LNPs containing 20 μg OVA mRNA (n = 3 per group) (a-b) Flow cytometric analysis of IL-12 p70 in migratory cDC2s in dLNs. (c-d) Flow cytometric analysis of IL-12 p70 in resident cDC2s in dLNs. (e-f) Flow cytometric analysis of IL-6 in migratory cDC2s in dLNs. (g-h) Flow cytometric analysis of IL-6 in resident cDC2s in dLNs. N.T., non-treated. Data are shown as means with SEM. Statistical significances were determined by one-way ANOVA with Dunnett’s *post hoc* tests. *, P < 0.05. **, P < 0.01. ****, P < 0.0001.

**
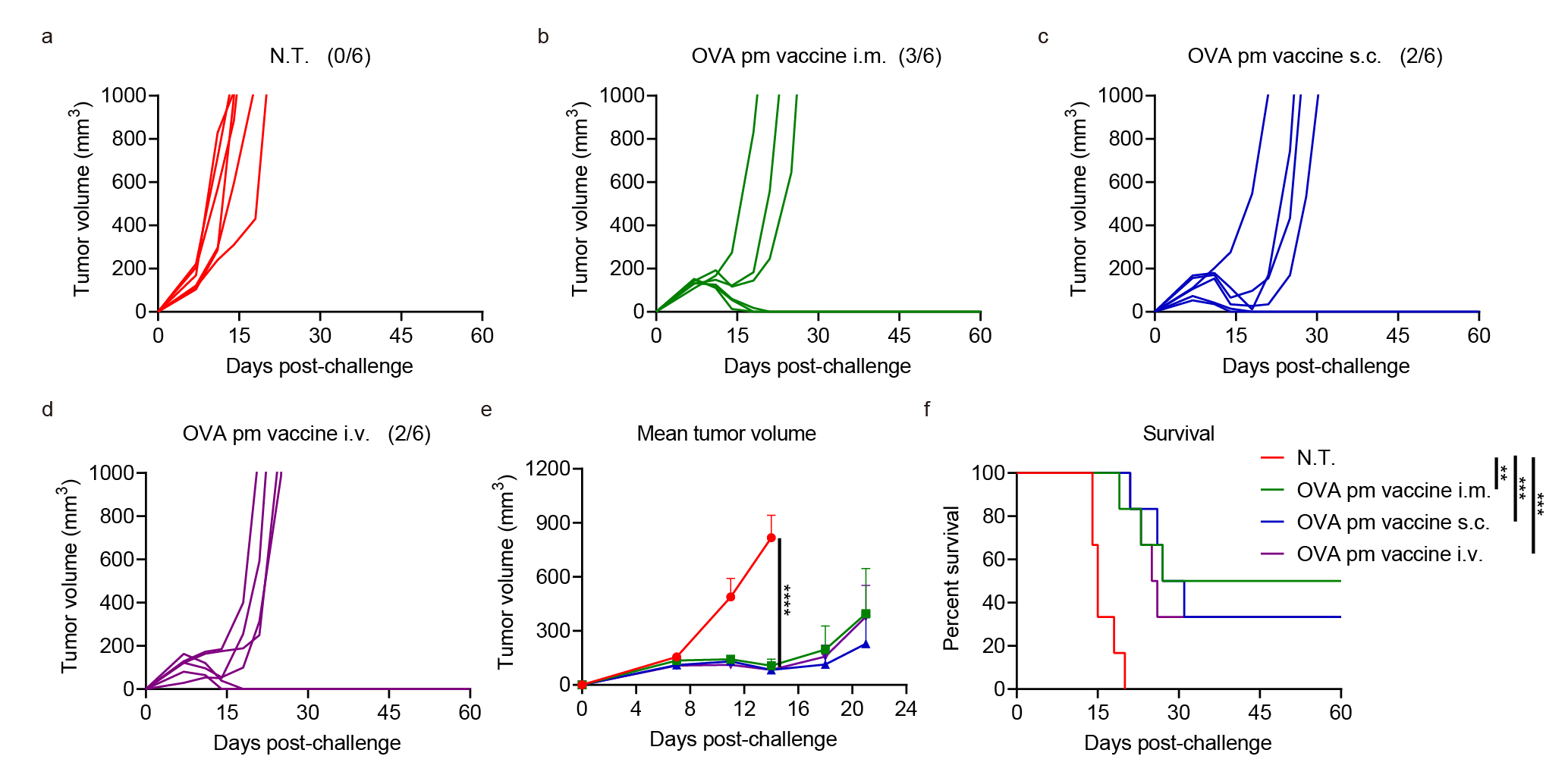
Figure S5. Therapeutic efficacies of vaccination via different routes on established E.G7-OVA tumors.** C57BL/6 mice were challenged s.c. on day 0 with 3×10^5^ E.G7-OVA cells, then immunized c.l. on day 3 with an intramuscular (i.m.), subcutaneous (s.c.) or intravenous (i.v.) dose of mRNA vaccines containing 20 μg OVA mRNA (n = 6 per group), respectively. (a-d) Individual growth curves of E.G7-OVA tumors in mice in therapeutic assay. Fractions in the parentheses indicate the proportion of cured mice in the groups. (e) Mean volumes of E.G7-OVA tumors in mice in therapeutic assay. Data are shown as means with SEM. Statistical significances were determined by one-way ANOVA with Dunnett’s *post hoc* tests. (f) Survival curves of mice bearing E.G7-OVA tumors in therapeutic assay. Statistical significances were determined by log-rank test. **, P < 0.01. ***, P < 0.001. ****, P < 0.0001.

**
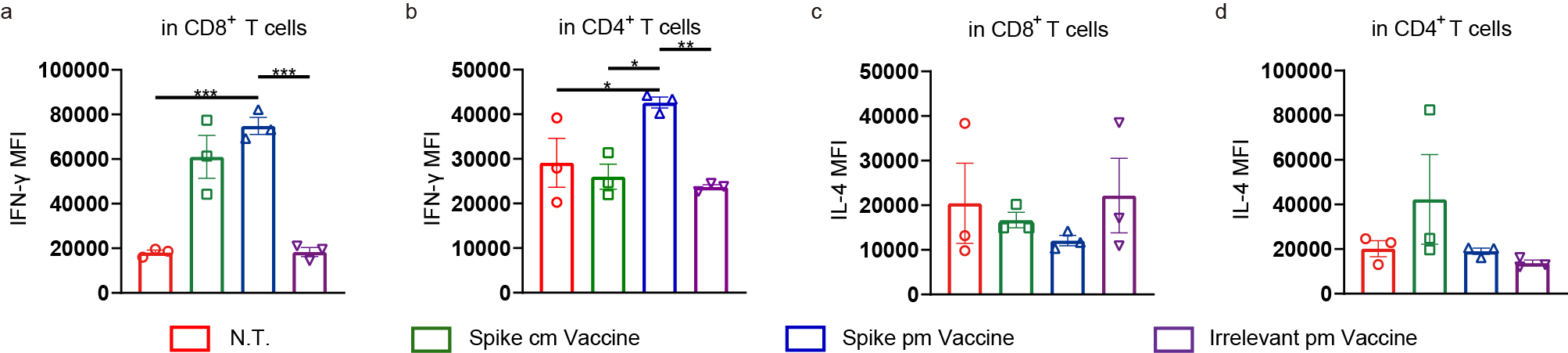
**

**Figure S6. Flow cytometric analysis of expression levels of intracellular cytokines in restimulated splenic T cells after Spike Vaccination.** Splenocytes were restimulated by peptide pool spanning SARS-CoV-2 spike protein and stained for intracellular cytokines. (a) Mean fluorescence intensities (MFIs) of IFN-γ staining in IFN-γ^+^CD8^+^ T cells. (b) MFIs of IFN-γ staining in IFN-γ^+^CD4^+^ T cells. (a) MFIs of IL-4 staining in IL-4^+^CD8^+^ T cells. (a) MFIs of IL-4 staining in IL-4^+^CD4^+^ T cells. N.T., non-treated. Data are shown as means with SEM. Statistical significances were determined by one-way ANOVA with Dunnett’s *post hoc* tests. *, P < 0.05. **, P < 0.01. ***, P < 0.001.

| Number | Gene | Neoantigen peptide | Substitution | Responder T cell type |
| --- | --- | --- | --- | --- |
| 1 | Aldh18a1 | LHSGQNHLKEMAISVLEARACAAAGQS | P154S | CD4^+^ |
| 2 | Als2 | GYISRVTAGKDSYIALVDKNIMGYIAS | L675I | CD8^+^ |
| 3 | Nphp3 | AGTQCEYWASRALDSEHSIGSMIQLPQ | G234D | CD4^+^ |
| 4 | Dkk2 | EGDPCLRSSDCIDEFCCARHFWTKICK | G192E | CD4^+^ |
| 5 | Steap2 | VTSIPSVSNALNWKEFSFIQSTLGYVA | R388K | CD4^+^ |
| 6 | Slc4a3 | PLLPFYPPDEALEIGLELNSSALPPTE | T373I | CD4^+^ |
| 7 | E2f8 | VILPQAPSGPSYATYLQPAQAQMLTPP | I522T | CD8^+^ |
| 8 | Slc20a1 | DKPLRRNNSYTSYIMAICGMPLDSFRA | T425I | CD4^+^ |
| 9 | Dhx35 | EVIQTSKYYMRDVIAIESAWLLELAPH | T646I | CD4^+^ |
| 10 | Agxt2l2 | EHIHRAGGLFVADAIQVGFGRIGKHFW | E247A | CD4^+^ |

**Table S1.** **CT26 neoantigens used in this study.** The information was cited from Kreiter, S., et al., *Mutant MHC class II epitopes drive therapeutic immune responses to cancer.* Nature, 2015. **520**(7549): p. 692-6.
